# Active Task Engagement Enhances Auditory Brain–Behaviour Prediction From Single‐Trial EEG Compared With Passive Listening

**DOI:** 10.1111/ejn.70438

**Published:** 2026-02-20

**Authors:** Zhaonan Ma, Xiaoyu Wang, Xiao Yang, Chao Guo, Tommi Kärkkäinen, Fengyu Cong

**Affiliations:** ^1^ School of Biomedical Engineering, Faculty of Medicine Dalian University of Technology Dalian China; ^2^ Faculty of Information Technology University of Jyväskylä Jyväskylä Finland; ^3^ Western Institute of Neuroscience Western University London Canada; ^4^ Department of Physiology and Pharmacology Western University London Canada; ^5^ Key Laboratory of Social Computing and Cognitive Intelligence Dalian University of Technology China; ^6^ School of Software Engineering Dalian University Dalian China

**Keywords:** brain–behaviour coupling, event‐related potentials (ERPs), mismatch negativity (MMN), single‐trial EEG decoding

## Abstract

Auditory neural processing during active task engagement and passive listening reflects distinct task contexts with potentially different behavioural relevance. While both contexts elicit deviance‐related responses, it remains unclear, which yields neural measures that more reliably predict individual differences in behavioural performance. To address this question, we employed a multi‐feature auditory paradigm in which frequency, duration, and intensity deviants were presented under passive (no response required) and active (explicit detection required) conditions. EEG was recorded from 47 participants; passive listening was characterized by a prominent mismatch negativity (MMN), whereas active discrimination was characterized by an additional P3b component. Beyond conventional ERP measures, we quantified individual‐level neural discriminability using EEGNet, a neural‐network–based classifier, by classifying deviant versus standard single‐trial epochs and deriving cross‐validated decoding accuracy. Behavioural performance was quantified using an efficiency score (ES) that integrates hit rate and reaction time. Participants were stratified into high‐ and low‐performance groups based on a median split of ES. Results showed that the expected MMN during passive listening and the P3b during active discrimination were elicited, as confirmed by spatiotemporal cluster‐based permutation analysis. Furthermore, decoding accuracy derived from the active discrimination condition robustly separated high‐ and low‐performance groups (Group × Task: F = 29.62, *p* < 0.001) and predicted behavioural efficiency across individuals (r = 0.53, *p* < 0.01). In contrast, passive‐listening decoding showed reduced overall discriminability and minimal group separation. Together, these findings indicate that task engagement amplifies the behavioural relevance of single‐trial neural discriminability, enabling stronger auditory brain–behaviour prediction than passive listening.

## Graphical abstract

This study investigates whether neural responses obtained under different task contexts provide reliable predictors of behavioural performance in an auditory discrimination task. Using EEGNet to classify single‐trial event‐related potentials, we analysed neural discriminability between standard and deviant stimuli during active task engagement and passive listening. The results demonstrate that classification accuracy derived from the active task significantly predicts individual efficiency scores (ES), whereas classification accuracy derived from passive listening shows no predictive value. These findings indicate that neural representations expressed during active task engagement provide a more robust and behaviourally informative predictor of performance than neural responses obtained during passive listening.

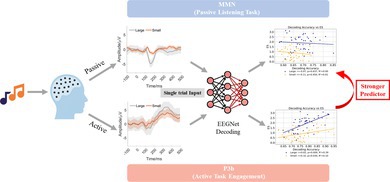

**Abbreviations**
ANOVA: analysis of variance
EEG: electroencephalography
ERPs: event‐related potentials
ES: efficiency score
ICA: Independent component analysis
MMN: mismatch negativity
RT: reaction time
TFCE: threshold‐free cluster enhancement

## 1 Introduction

Auditory deviance processing is often examined across task contexts, typically defined by whether listeners are required to detect and respond to sound changes, spanning passive listening and active discrimination tasks (Dinces and Sussman 2017). These contexts can shape how acoustic information is prioritized in complex auditory environments (Shinn‐Cunningham 2008). During active tasks, overt behavioural requirements bias processing toward task‐relevant sounds, enhancing target‐stream representations relative to competing background input (Fritz et al. 2003). This prioritization reflects the brain's limited capacity to process competing auditory streams and is known to improve discrimination performance under demanding listening conditions (Hillyard et al. 1973; Choi et al. 2013, 2014). In contrast, passive listening paradigms require no overt responses and are commonly used to probe auditory processing when no explicit decision or response to sound changes is required. Although both active and passive paradigms provide valuable insights into auditory deviance processing (Laffere et al. 2020), it remains unclear whether neural responses elicited under these task contexts show comparable predictive value for individual differences in behavioural performance.

Quantifying behavioural performance in a way that is comparable across individuals is critical for interpreting brain–behaviour relationships. Behavioural performance is typically quantified by reaction time (RT) and accuracy (hit rate), which index complementary aspects of task execution and can be differentially affected by attentional and decisional factors (Prinzmetal et al. 2005; Van Ede et al. 2012; Mulder and Van Maanen 2013). However, reliance on either measure alone may yield an incomplete characterization of overall task efficiency, particularly when speed–accuracy trade‐offs differ across individuals or task contexts (Bruyer and Brysbaert 2011; Liesefeld and Janczyk 2019). Accordingly, composite measures that integrate response speed and accuracy may provide a more suitable index of overall task efficiency for individual‐differences analyses.

At the neural level, electroencephalography (EEG) is well suited for characterizing the temporal dynamics of auditory processing due to its high temporal resolution. In auditory oddball paradigms, event‐related potentials (ERPs) are widely used to index deviance processing, most notably mismatch negativity (MMN) and P3b (Boucher et al. 2010; Wang et al. 2025). MMN is robustly elicited in passive listening paradigms and is commonly interpreted as an early index of neural sensitivity to violations of acoustic regularities (Sussman et al. 2014; Wang et al. 2024). Its amplitude and latency can be modulated by attentional state and task requirements, and some studies have linked individual variation in MMN to behavioural performance in auditory discrimination tasks (Näätänen et al. 2007; Pakarinen et al. 2007). In contrast, the P3b is most reliably elicited by task‐relevant targets during active discrimination and has been associated with task engagement and updating/decision‐related processes, with systematic modulation by task demands and performance accuracy (Polich 2007; Golob and Holmes 2011). Together, MMN and P3b offer complementary indices of deviance processing under passive versus active task contexts, motivating the question of whether these context‐specific neural responses differ in their relevance for individual differences in behaviour.

Although the MMN and P3b components are both widely used as neural markers of auditory deviance processing, accumulating evidence suggests that they show limited cross‐paradigm equivalence and may reflect distinct underlying mechanisms (Fitzgerald and Todd 2020; Verleger 2020). For example, related works directly compared traditional MMN and P3b paradigms with a local–global sequence and demonstrated that, while both approaches reliably elicited the expected local/global and MMN/P3b responses at the group level, their within‐individual sensitivity and cross‐paradigm comparability differed (Bekinschtein et al. 2009; Rutiku et al. 2024). Specifically, the P3b exhibited strong amplitude correlations across paradigms, consistent with a relatively stable, task‐relevant target‐processing component, whereas the so‐called local effect, often assumed to index MMN, differed markedly from the canonical MMN in its timing, morphology, and relationship to subsequent P3a activity (Rutiku et al. 2024). These findings indicate that MMN and P3b capture complementary but not interchangeable aspects of auditory deviance processing, and further suggest that neural responses obtained under passive listening and active task engagement may differ in how well they predict individual differences in overall behavioural efficiency. Despite extensive work examining the functional roles of MMN and P3b separately (Fogarty et al. 2019; Fong et al. 2020), few studies have directly compared their predictive value for behavioural performance across task contexts, particularly when using single‐trial neural indices of discriminability.

To complement conventional ERP measures, it is important to characterize deviance‐related activity at the single‐trial level, rather than relying solely on averaged MMN/P3b amplitude and latency indices. Although ERP measures remain the standard approach for characterizing these components, they provide only a partial characterization of the trial‐level discriminability between deviant‐ and standard‐evoked EEG responses. Accordingly, multivariate classification approaches—including machine learning and deep learning methods—have been increasingly adopted to complement traditional ERP analyses, because they can quantify single‐trial decodability even when differences are subtle in averaged waveforms (Craik et al. 2019; Roy et al. 2019). Deep learning architectures such as EEGNet are well suited for this purpose, as they can learn task‐relevant spatiotemporal filters from minimally processed epoched EEG and reduce reliance on handcrafted feature extraction and a priori feature selection (Lawhern et al. 2018). By providing an index of how reliably deviant‐related activity can be distinguished from standard activity on single trials, such approaches offer a principled way to relate neural discriminability to individual differences in behavioural efficiency.

This study investigates whether neural responses elicited during active task engagement versus passive listening differ in their ability to predict individual differences in auditory discrimination performance. We operationalize neural discriminability at the single‐trial level as cross‐validated decoding accuracy for classifying deviant versus standard event‐related EEG epochs, indexing how distinctly deviance‐related information is represented in the EEG signal. To manipulate deviance magnitude and create a graded range of perceptual salience, we included both small and large deviants. Accordingly, we formulated two directional hypotheses. First, we hypothesized higher deviant‐versus‐standard single‐trial decoding accuracy in the active task than in passive listening, consistent with enhanced discriminability under explicit behavioural demands. We further expected higher decoding accuracy for large than for small deviants, given their greater perceptual salience. Second, we hypothesized that active‐task neural measures, particularly single‐trial discriminability indices, would show stronger brain–behaviour associations with individual differences in behavioural efficiency than analogous measures derived from passive listening. Together, these hypotheses test whether active‐task neural responses provide greater predictive value for auditory discrimination performance than passive‐listening responses, when behavioural outcomes integrate both response speed and accuracy.

## 2 Materials and Methods

### 2.1 Participants

The study enrolled 47 male participants (age range: 21–29 years; mean age 25 ± 2.42 years). All participants were right‐handed, had normal or corrected‐to‐normal vision, and had no history of neurological or psychiatric disorders. Ethical approval was obtained from the Biomedical Engineering Ethics Committee of Dalian University of Technology (DUTFM241121‐03), and all participants provided written informed consent.

### 2.2 Auditory Materials and Procedure

Auditory stimuli were adapted from the well‐established multi‐feature Optimum‐1 paradigm (Näätänen et al. 2004; Pakarinen et al. 2007). Standard tones consisted of three sinusoidal partials (523, 1046, and 1569 Hz), with the fundamental corresponding to C5; the second and third partials were presented 3 and 6 dB lower than the fundamental, respectively. All tones were 75 ms in duration, including 5 ms rise/fall times, and were delivered binaurally via headphones at 60 dB SPL for all participants. Three types of deviant stimuli were employed (frequency, duration, and intensity), each presented at two deviance levels (small and large). The corresponding stimulus parameters are summarized in Table 1.

**TABLE 1:** Stimulus parameters for standard, small‐deviant, and large‐deviant conditions.

Stimulus level	Duration (ms)	Frequency (Hz)	Intensity (dB)
Standard	75	523	60
Small deviant	67	527	57.5
Large deviant	43	562	50

**FIGURE 1:**
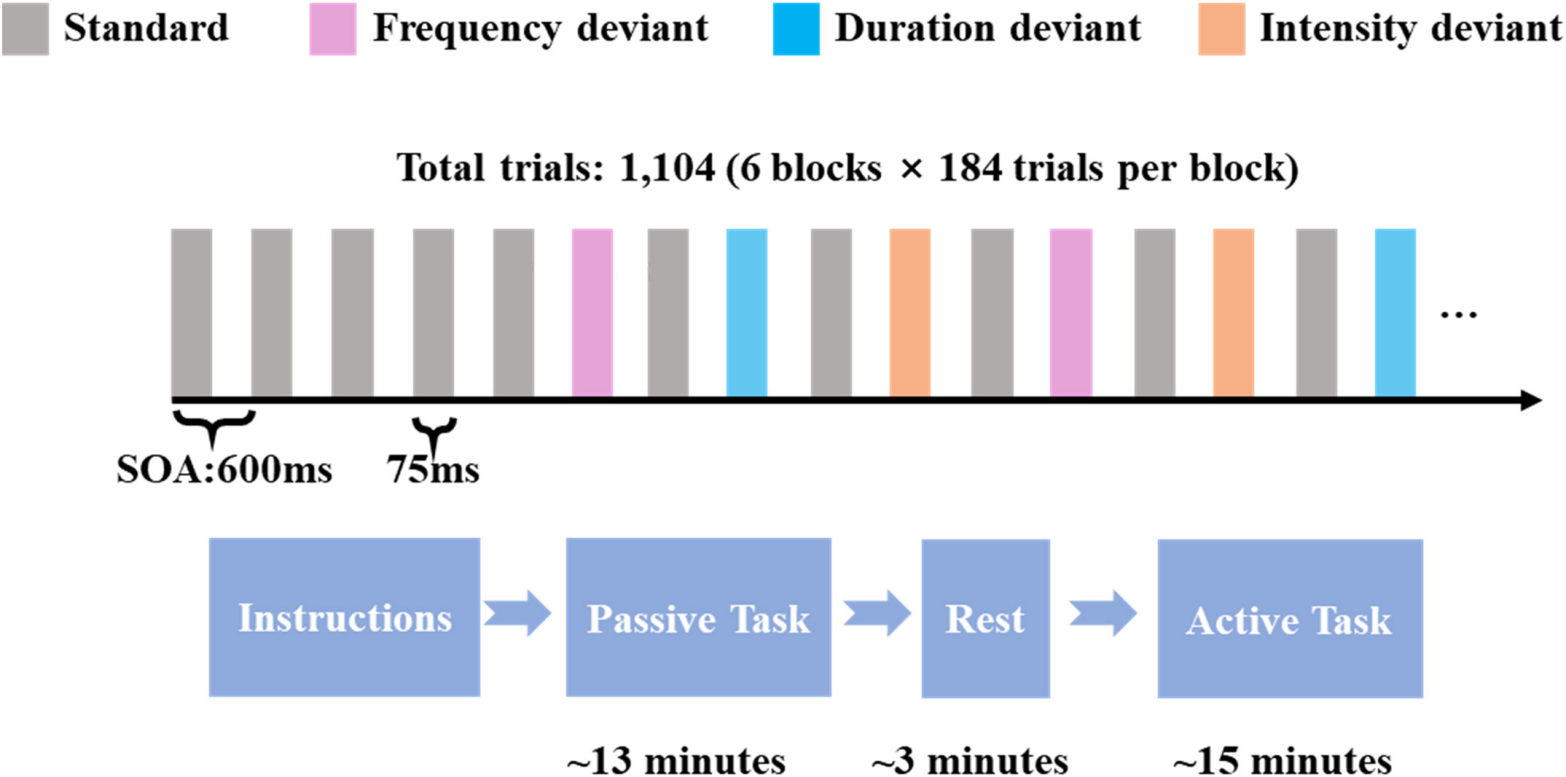
Schematic illustration of the auditory multi‐feature oddball paradigm and experimental procedure. Auditory sequences consisted of frequent standard tones (grey) interspersed with frequency (pink), duration (blue), and intensity (orange) deviants presented in a pseudo‐random order. Each block comprised 184 trials (total = 1104 trials across six blocks) with a stimulus onset asynchrony (SOA) of 600 ms and a stimulus duration of 75 ms. Participants first completed a passive listening task (~13 min) without making any overt responses while watching a silent movie. After a brief rest (~3 min), participants performed an active auditory discrimination task (~15 min) using the same stimulus sequence, during which they responded by pressing the spacebar whenever the task‐relevant deviant occurred.

Stimuli were presented in six blocks (Figure 1). Each block contained 184 trials, starting with four standards, followed by an alternating standard–deviant sequence (i.e., each deviant was preceded by a standard). Deviant types (frequency, duration, intensity) were pseudo‐randomized such that no two consecutive deviants were of the same type. Across all blocks, the paradigm comprised 1104 trials, including 564 standards and 540 deviants. Deviants were balanced across deviant types (180 each) and magnitudes, with each type comprising 90 large deviants (LD) and 90 small deviants (SD).

The experiment consisted of two tasks: a passive listening task followed by an active auditory discrimination task, both using the identical stimulus sequence. During the passive listening task, auditory stimuli were presented across six blocks (~13 min) while participants watched a silent movie and made no overt responses to divert attention away from the sounds and maintain a consistent passive context. After a brief rest period, the same stimulus sequence was presented in an active auditory discrimination task, in which participants responded only to task‐relevant deviants while ignoring other deviant types. Specifically, the response rule changed every two blocks (frequency, duration, then intensity), and participants pressed the spacebar only for the currently instructed target deviant type, with brief breaks between segments to deliver updated instructions. Stimulus presentation and response collection were implemented in MATLAB (R2022a) using Psychophysics Toolbox Version 3 (Brainard 1997). Hit rates and RTs were recorded during the active task.

### 2.3 Behavioural Measurement

Behavioural analyses focused on responses to target (task‐relevant deviant) stimuli occurring within 200–1000 ms post‐onset; responses outside this window were not counted as valid detections. For each participant, hit rate (proportion of targets detected within the window) and mean RT for correct detections were computed separately for LD and SD in the active discrimination task, as no overt responses were collected during passive listening. An efficiency score (ES) was additionally computed as ES = hit rate/RT, with higher values indicating more efficient performance. Hit rate and RT were reported as descriptive statistics in the Results, whereas brain–behaviour analyses primarily used ES as the behavioural index.

For group‐level comparisons, participants were divided into high‐ES and low‐ES groups based on the median ES. Group differences in neural decoding performance were then tested between the two groups. In addition, regression analyses treated ES as a continuous outcome to quantify associations between neural measures and behavioural performance across participants.

### 2.4 EEG Recordings and Preprocessing

Scalp EEG was recorded using an ANT Neuro amplifier with a 64‐channel Ag/AgCl cap positioned according to the extended 10–20 system. Data were acquired at 1000 Hz with an online reference at CPz and an online band‐pass filter of 0.1–100 Hz. Continuous EEG was preprocessed in EEGLAB (Delorme and Makeig 2004). Periods containing gross artefacts (e.g., body movements, amplifier clipping, or high‐amplitude bursts) were identified by visual inspection and removed. Data were then down‐sampled to 500 Hz, notch‐filtered at 50 Hz, and band‐pass filtered at 1–30 Hz. EEG was re‐referenced to the average of the left and right mastoids (M1 and M2). ICA was performed using the Infomax algorithm (Lee et al. 1999) to remove components reflecting eye blinks, horizontal eye movements, muscle activity, and ECG artefacts. Artefact‐related components were first classified using ICLabel (Pion‐Tonachini et al. 2019) and then manually verified. Channels exhibiting excessive artefacts were interpolated using spherical splines (Perrin et al. 1989). Finally, data were epoched from −100 to 500 ms relative to stimulus onset and baseline‐corrected using the −100 to 0 ms interval.

### 2.5 Data Analysis

#### 2.5.1 ERP Computation and Component Identification

ERPs are shown at Fz for visualization. This electrode was selected as a common viewing site across tasks where MMN is robust and P3b can also be readily observed, enabling direct comparison between the two components. For each participant, standard and deviant ERPs were computed separately by averaging trials within each deviant feature type (frequency, duration, and intensity) and deviance magnitude (LD and SD). Difference waves were then calculated by subtracting the standard waveform from the deviant waveform. MMN and P3b were identified in the difference waves within predefined latency windows that were time‐locked to the point of deviation in the stimulus: MMN as the most negative deflection within 150–200 ms during passive listening, and P3b as the most positive deflection within 250–500 ms during active discrimination.

#### 2.5.2 Statistical Validation of ERP Effects

Statistical significance of the difference waves was assessed using spatiotemporal cluster‐based permutation testing with threshold‐free cluster enhancement (TFCE) implemented in MNE‐Python (Maris and Oostenveld 2007; Sassenhagen and Draschkow 2019). Tests were performed on participant‐level averaged difference waves across the full sensor × time grid, including one‐sample *t*‐tests against zero within each task (passive listening; active discrimination) and paired *t*‐tests for the within‐subject comparison between tasks (active vs. passive). Spatiotemporal adjacency was defined using the sensor adjacency matrix derived from the 10–20 electrode montage. Statistical significance was evaluated with 1000 permutations controlling the family‐wise error rate at α = 0.05 (two‐tailed). For descriptive purposes, effects were summarized only when they involved at least two spatially adjacent electrodes and persisted for at least 20 ms. In the Results section, we report the onset and offset (ms) of significant clusters as illustrated at Fz to facilitate visualization and cross‐component comparability.

#### 2.5.3 Single‐Trial EEG Decoding With EEGNet

Single‐trial EEG decoding was performed with EEGNet (Lawhern et al. 2018). Specifically, we implemented EEGNet‐8,2 (F1 = 8, D = 2; F2 = 16), with a temporal kernel length of 64, a separable convolution kernel length of 16, average pooling of 4 and 8, dropout = 0.5 (Dropout), ELU nonlinearities, and batch normalization. For each participant, single‐trial EEG from 62 scalp electrodes (excluding the mastoids, M1 and M2) was used as input. Classification analyses were performed separately for each deviant type (frequency, duration, and intensity). Within each deviant type, two binary classifications were performed: LD versus standards and SD versus standards. For each deviant trial, the corresponding standard was defined as the immediately preceding standard trial, consistent with the stimulus‐pair structure of the paradigm. Accordingly, each classifier was trained on equal trial counts for the standard and deviant classes, thereby avoiding class‐imbalance effects.

Classification was conducted at the single‐participant level using 4‐fold cross‐validation separately for each condition (task × deviant type × deviance magnitude). For each participant and condition, trials were stratified and randomly partitioned into four folds: three for training and one held‐out fold for evaluation. Test accuracy was averaged across the four held‐out folds to obtain a single decoding accuracy for each participant and condition, which was used as an index of single‐trial neural discriminability in subsequent brain–behaviour analyses.

#### 2.5.4 Statistical Analysis of Decoding Accuracy and Behavioural Performance

Group‐level effects on decoding accuracy were tested using a mixed‐design ANOVA, with performance group (high vs. low; median split on behavioural ES) as a between‐subjects factor and two within‐subjects factors: task (active vs. passive) and deviant type (frequency, duration, intensity). Omnibus tests were used to assess main effects and interactions, and Tukey's HSD procedure (FWER = 0.05) was applied to follow‐up pairwise comparisons where appropriate.

In addition, linear regression analyses tested whether neural discriminability (single‐trial decoding accuracy) was associated with individual differences in behavioural efficiency (ES). For each deviant type, two separate models were fit with ES as the outcome and either LD‐versus‐standard or SD‐versus‐standard decoding accuracy as the predictor.

#### 2.5.5 Power Analysis

Sensitivity power analyses were conducted in G*Power 3.1 (Faul et al. 2007, 2009) using α = 0.05 (two‐tailed) and 1 − β = 0.80 to determine the minimum detectable effects given the final sample size (*N* = 47). For the between‐group comparison defined by the median split (high vs. low; *n* = 24 vs. *n* = 23), the minimum detectable effect was ηp^2^ = 0.15. For regression analyses relating EEGNet decoding accuracy to ES in a single‐predictor model, the minimum detectable association was |r| = 0.38.

## 3 Results

### 3.1 Behavioural Performance Across Deviance Types and Magnitudes

**FIGURE 2:**
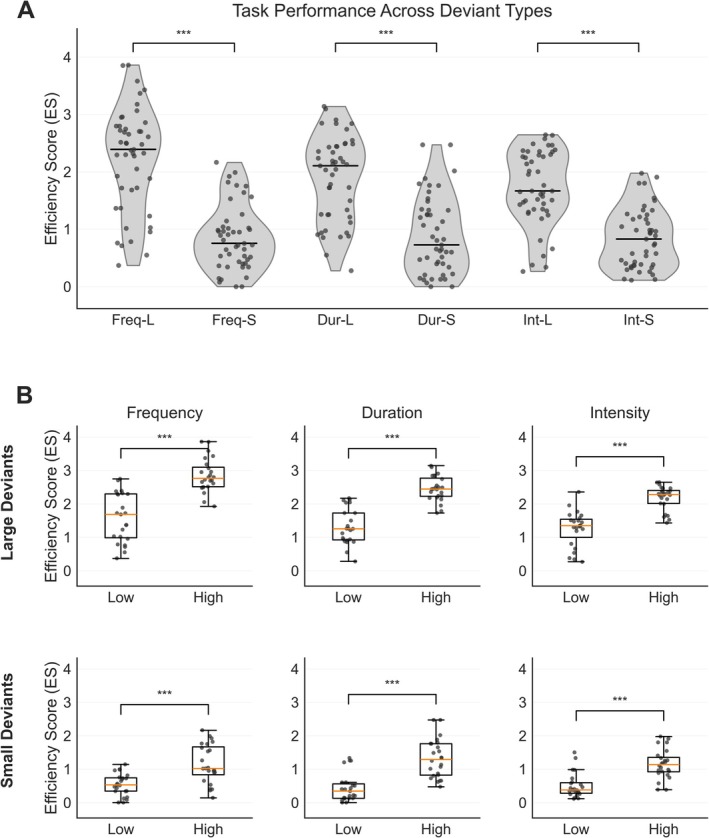
Behavioural efficiency across deviant types and performance groups. (A) Violin plots depict the distribution of ES across deviant types (frequency, duration, and intensity) and deviance magnitudes (large and small). Individual dots represent single participants, and horizontal bars denote medians. Across all three feature dimensions, ES values were significantly higher for large than for small deviants (***p < 0.001), indicating enhanced behavioural efficiency for more salient stimulus changes. (B) Boxplots illustrate ES distributions for low‐ and high‐performance groups, defined by a median split of composite ES scores, shown separately for each deviant type (columns) and deviance magnitude (rows). Within each deviant type, the high‐performance group consistently exhibited higher ES than the low‐performance group for both large and small deviants (***p < 0.001).

Behavioural results (as shown in Table 2) confirmed a robust manipulation of deviance magnitude: across deviant types, LD was associated with higher detection accuracy and shorter response times than SD, producing substantially higher behavioural efficiency (ES; *p* < 0.001). This consistent LD–SD separation confirmed that discrimination demands varied in the intended direction across feature types. Figure 2A presents the resulting ES distributions across deviant types and magnitudes, and Figure 2B shows that this efficiency gradient was preserved when stratifying participants by overall performance, with the high‐performance group exhibiting higher ES than the low‐performance group for both LD and SD across deviant types (all *p* < 0.001).

**TABLE 2:** Behavioural measurements for large and small deviants across deviant types.

Deviant type	Deviant level	Reaction time (ms)		Hit rate		ES	
		Mean	s.d.	Mean	s.d.	Mean	s.d.
Frequency	Large	380	60	0.81	0.25	2.23	0.89
	Small	490	60	0.41	0.26	0.85	0.56
Duration	Large	440	80	0.80	0.23	1.89	0.74
	Small	500	50	0.43	0.30	0.89	0.67
Intensity	Large	460	60	0.77	0.24	1.73	0.65
	Small	480	50	0.40	0.23	0.84	0.52

### 3.2 Task Effects on ERP Responses to Deviance

**FIGURE 3:**
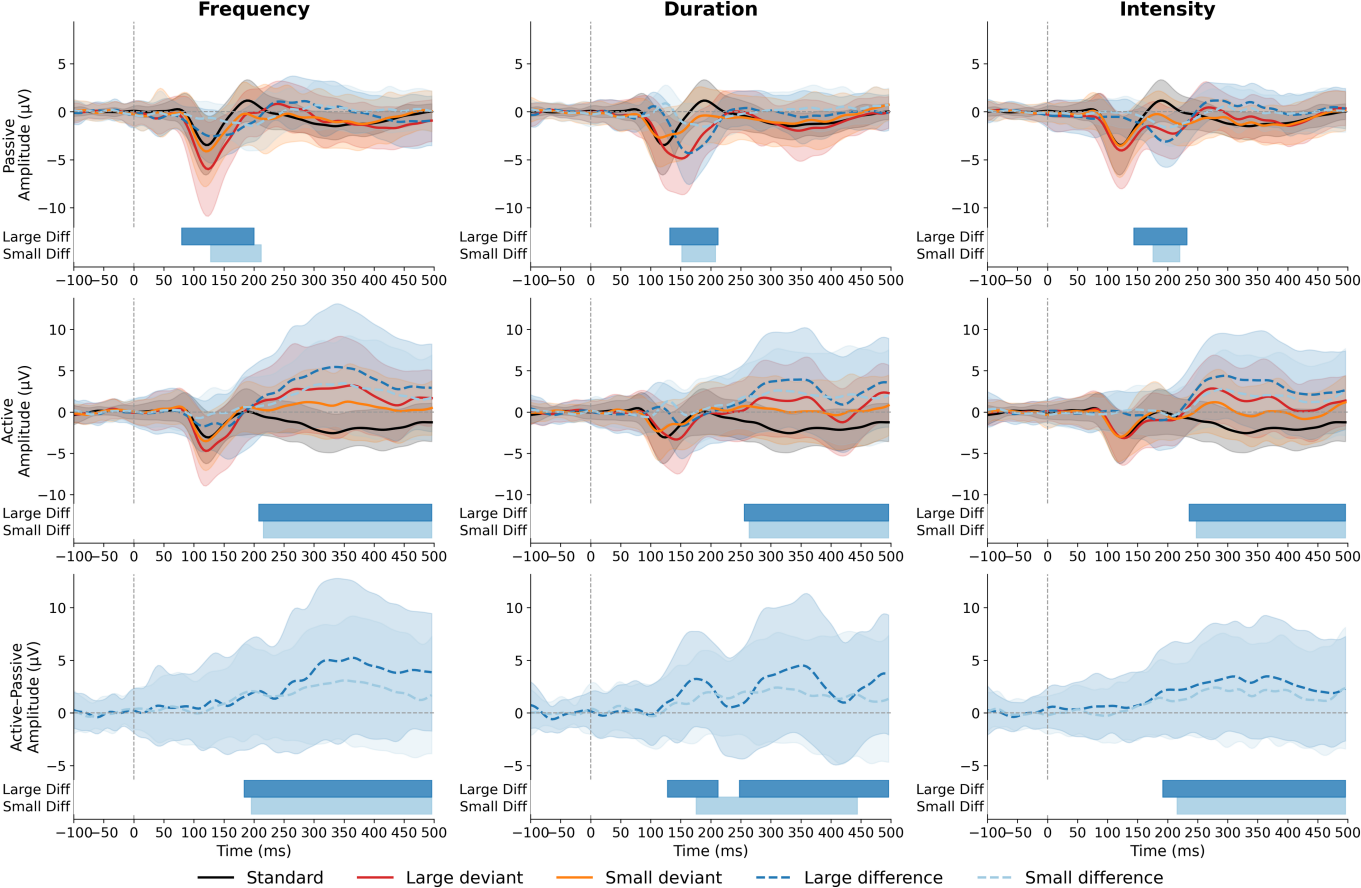
Grand‐averaged ERP waveforms (solid lines) and deviant–standard difference waves (dashed lines) at Fz are shown for frequency, duration, and intensity deviants under passive listening (top row) and active discrimination (middle row). The bottom row shows the active–passive contrast of the difference waves (active minus passive). Solid lines depict responses to standards (black), large deviants (red), and small deviants (orange); dashed lines depict the corresponding difference waves for large (dark blue) and small (light blue) deviants. Shaded envelopes indicate ±1 SD across participants. Horizontal bars beneath each panel mark significant time intervals identified by spatiotemporal cluster‐based permutation tests: in the top and middle rows, bars indicate periods when the large‐ or small‐deviant difference wave significantly deviated from zero; in the bottom row, bars indicate periods when active and passive difference waves differed significantly, reflecting task‐dependent modulation of deviance processing.

At the group level, spatiotemporal cluster‐based permutation tests on difference waves revealed robust, task‐dependent ERP responses to deviance across feature types (frequency, duration, intensity) and magnitudes (large, small) (Figure 3; all cluster‐wise *p* < 0.05).

During passive listening, difference waves showed significant early negative deflections consistent with MMN across all deviant types and magnitudes. Significant clusters were observed for frequency deviants (LD: 80–200 ms, peak t = 8.06 at 156 ms; SD: 128–212 ms, peak t = 4.24 at 160 ms), duration deviants (LD: 132–212 ms, peak t = 9.45 at 184 ms; SD: 152–208 ms, peak t = 5.48 at 188 ms), and intensity deviants (LD: 144–232 ms, peak t = 8.01 at 196 ms; SD: 176–220 ms, peak t = 6.13 at 192 ms).

During active discrimination, significant clusters reflected a pronounced late positive deflection consistent with P3b across all deviant types and magnitudes. For frequency deviants, significant positivity spanned 208–496 ms for LD (peak t = 5.01 at 468 ms) and 216–496 ms for SD (peak t = 4.54 at 336 ms). Similar late effects were observed for duration deviants (LD: 256–496 ms, peak t = 4.67 at 332 ms; SD: 264–496 ms, peak t = 3.54 at 332 ms) and intensity deviants (LD: 236–496 ms, peak t = 5.85 at 296 ms; SD: 248–496 ms, peak t = 3.57 at 336 ms).

Direct comparisons between tasks (active vs. passive) showed that between‐condition effects were concentrated mainly in the late interval, indicating enhanced late positivity under active engagement. For frequency deviants, significant late clusters were observed for LD (184–496 ms, peak t = 5.01 at 468 ms) and SD (196–496 ms, peak t = 3.77 at 344 ms). For intensity deviants, comparable late between‐condition effects were present for LD (192–496 ms, peak t = 4.56 at 304 ms) and SD (216–496 ms, peak t = 3.66 at 312 ms). For duration deviants, LD showed two significant clusters—an earlier cluster at 128–212 ms and a late cluster at 248–496 ms (peak t = 4.81 at 340 ms)—whereas SD showed a significant late cluster at 176–496 ms (peak t = 4.02 at 348 ms).

### 3.3 EEGNet Model Decoding Accuracy for Deviant vs. Standard Stimuli

**FIGURE 4:**
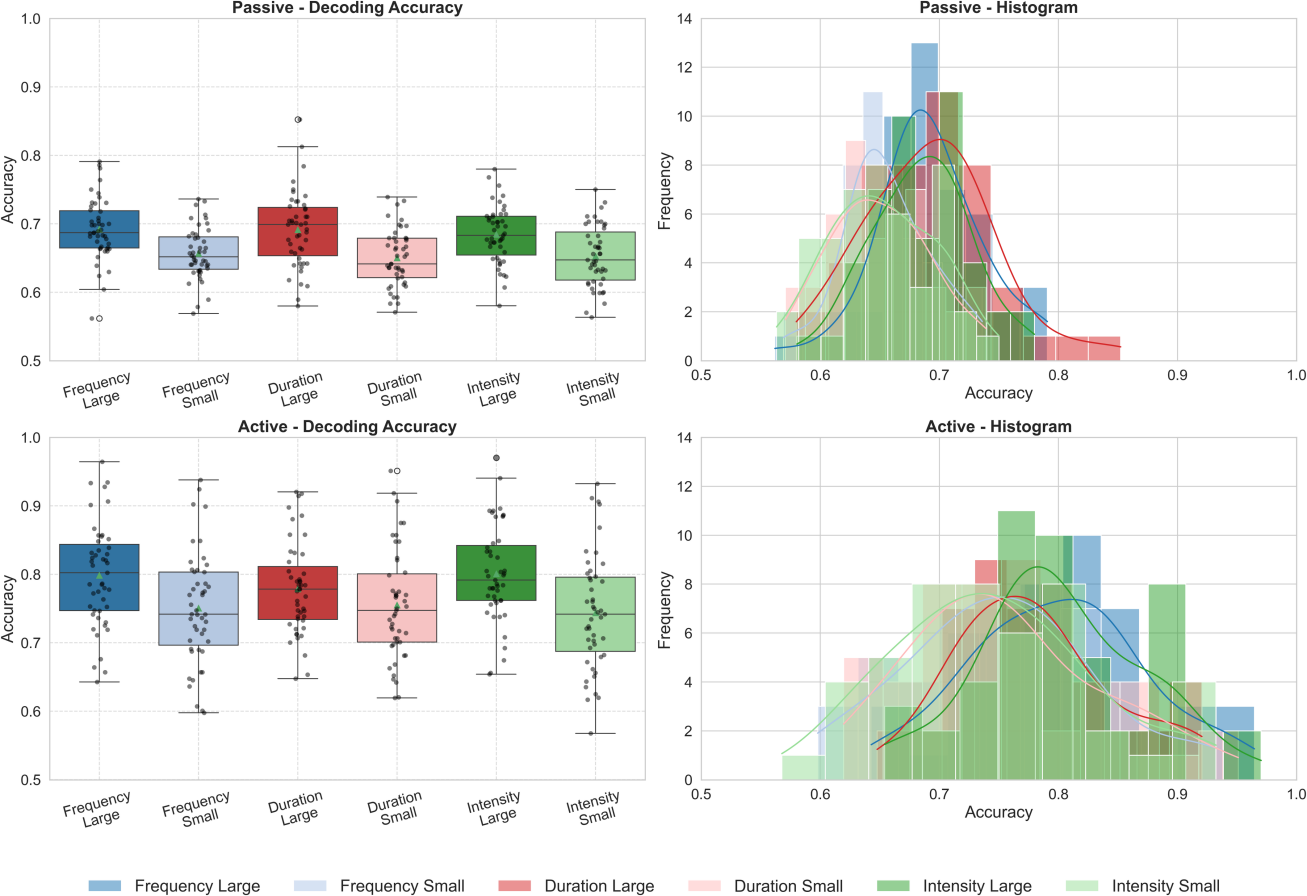
Single‐trial decoding accuracy for large (LD) and small (SD) deviants across tasks and deviant types. Boxplots (left) show participant‐level decoding accuracy for frequency, duration, and intensity deviants (LD and SD each, vs. standards) in the passive condition (top) and active condition (bottom); overlaid points indicate individual participants. Histograms (right) show the corresponding accuracy distributions for each deviant type and magnitude (colour‐coded).

As shown in Figure 4, decoding accuracy for classifying deviant versus standard trials was consistently higher for LD than for SD in both tasks. In the passive condition, decoding accuracy (mean ± SD; *N* = 47) was 0.69 ± 0.05 (frequency), 0.69 ± 0.06 (duration), and 0.68 ± 0.04 (intensity) for LD, compared with 0.66 ± 0.04, 0.65 ± 0.04, and 0.65 ± 0.04 for SD. In the active condition, decoding accuracy increased overall, reaching 0.80 ± 0.08 (frequency), 0.78 ± 0.07 (duration), and 0.80 ± 0.07 (intensity) for LD, versus 0.75 ± 0.08, 0.76 ± 0.08, and 0.74 ± 0.08 for SD. Distributional summaries paralleled the mean pattern, with higher accuracy in the active than passive condition. Decoding accuracies during passive listening were tightly clustered (median: LD = 0.68–0.70; SD = 0.64–0.65; IQR = 0.05–0.07). Under active discrimination, accuracies shifted upward and exhibited broader interquartile spreads (median: LD = 0.78–0.80; SD = 0.74–0.75; IQR = 0.08–0.11), suggesting both higher typical decoding performance and greater between‐participant variability with task engagement.

### 3.4 Group and Task Differences in Decoding Accuracy

Table 3 summarizes the results of the mixed‐design ANOVA on decoding accuracy with Group (high‐ vs. low‐performance), Task (active vs. passive), and Deviant Type (frequency, duration, intensity) as factors. The results showed significant main effects of Group (F = 41.29, *p* < 0.001) and Task (F = 369.01, *p* < 0.001), indicating higher decoding accuracy in the high‐performance than the low‐performance group and higher decoding accuracy in the active task than in the passive listening condition. A significant Group × Task interaction was also observed (F = 29.62, *p* < 0.001). Follow‐up Tukey HSD tests (FWER = 0.05) showed that, in the active condition, decoding accuracy was significantly higher in the high‐performance group than in the low‐performance group for all three deviant types (frequency and duration: *p* < 0.001; intensity: *p* = 0.0027). In contrast, in the passive condition, decoding accuracy was lower overall and high–low group differences were not significant for any deviant type (all *p* ≥ 0.936).

**TABLE 3:** The mixed‐design ANOVA results comparing decoding accuracy between the high‐ and low‐performance groups in both active and passive tasks with different deviant types.

Source	F	*p*	ηp^2^
Group	41.29	< 0.001	0.27
Deviant type	0.41	0.66	0.04
Task	369.01	< 0.001	0.82
Group × Deviant type	1.07	0.35	0.06
Group × Task	29.62	< 0.001	0.23
Deviant type × Task	0.17	0.85	0.02
Group × Deviant type × Task	0.30	0.74	0.03

### 3.5 Regression Analysis of Behavioural Efficiency and Decoding Accuracy

**FIGURE 5:**
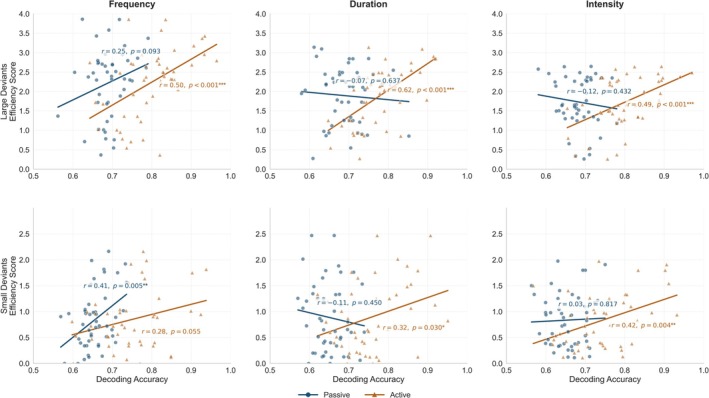
Scatterplots show the association between single‐trial EEGNet decoding accuracy (deviant vs. standard) and ES for frequency, duration, and intensity deviants (left to right). Results are shown separately for large (top row) and small (bottom row) deviants in the passive (blue) and active (orange) tasks. Regression lines are overlaid for each task, and in‐panel annotations report Pearson's r and corresponding *p* values. Across deviant types, decoding accuracy in the active task showed consistent positive associations with ES, particularly for large deviants, whereas passive‐task associations were weaker and less consistent.

As illustrated in Figure 5, decoding accuracy in the active task was positively associated with ES. For LD condition, correlations were significant for frequency (r = 0.50, *p* < 0.001), duration (r = 0.62, *p* < 0.001), and intensity (r = 0.49, *p* < 0.001). For SD condition, associations were weaker but remained significant for duration (r = 0.32, *p* = 0.030) and intensity (r = 0.42, *p* = 0.004), whereas the correlation for frequency did not reach significance (r = 0.28, *p* = 0.055). In contrast, decoding accuracy in the passive listening condition showed limited evidence of associations with behavioural efficiency. The only significant correlation was observed for SD in frequency deviance (r = 0.41, *p* = 0.005), whereas all remaining correlations for both deviance magnitudes (both LD and SD) in the passive condition were non‐significant (*p* values = 0.093–0.817). Because no overt responses were collected during passive listening, ES was computed exclusively from behavioural performance in the active task. Therefore, all brain–behaviour associations reported here relate neural measures from both the passive and active conditions to the same behavioural index derived from the active task.

## 4 Discussion

The present study shows that neural responses elicited during active task engagement are more predictive of individual differences in auditory discrimination performance than responses measured during passive listening. As expected from the deviance‐magnitude manipulation, larger deviants were associated with higher hit rates, faster responses, and greater behavioural efficiency, indicating a graded variation in discrimination difficulty. At the neural level, both tasks elicited expected deviance‐related responses, and single‐trial decoding indicated that deviant‐versus‐standard activity was decodable under both task contexts. Critically, decoding accuracy from the active task clearly differentiated high‐ versus low‐efficiency performers and predicted individual behavioural efficiency, whereas decoding based on passive listening showed limited predictive value. Together, these findings suggest that explicit task engagement yields neural representations that more closely track behavioural efficiency, highlighting the central role of task context in linking neural activity to individual differences in performance.

Behavioural performance showed a graded effect of deviance magnitude: compared with SD, LD yielded higher hit rates, faster responses, and greater ES. This pattern suggests that discrimination difficulty varied across conditions, with SD imposing higher demands than LD. Rather than implying a simple loss of sensitivity, reduced responding to SD may be consistent with greater uncertainty under low‐salience conditions (Quiroga‐Martinez et al. 2019). Importantly, ES captured these differences in a single continuous metric that integrates both RT and hit rate. Because ES summarizes overall discrimination efficiency, it is well suited for characterizing inter‐individual variability when speed–accuracy trade‐offs may differ across participants (Heitz 2014; Liesefeld and Janczyk 2019). This enabled a more transparent link between behavioural outcomes and variability in neural decoding, clarifying how neural discriminability relates to individual differences in auditory discrimination efficiency.

At the neural level, we focused on two prominent ERP components, MMN and P3b, which provide complementary indices of deviance‐related processing across passive and active task contexts. Prior work suggests that MMN is commonly associated with early sensitivity to acoustic change, whereas P3b is more closely linked to later evaluative processing of task‐relevant events (Fields 2023; Csépe and Honbolygó 2024). Although MMN has been linked to behavioural accuracy and response speed (Pakarinen et al. 2007), the strength of such links may be moderated by task demands and the behavioural relevance of deviant information. In contrast, P3b is most prominently expressed during active discrimination and has been proposed to reflect task‐relevant evaluation and response‐related decision processes (Verleger 2020). In the present study, contrasts between passive listening and active engagement illustrate how task context shapes the functional relevance of these responses. Deviance‐related responses during passive listening showed weaker/less consistent associations with individual differences in behavioural efficiency, whereas active‐task responses, particularly P3b‐related indices, were more strongly associated with performance. This pattern is consistent with accounts in which task engagement increases the behavioural relevance of sensory representations, strengthening brain–behaviour coupling under goal‐directed demands (Sherwell et al. 2017).

While both MMN and P3b provide valuable information about auditory deviance processing, they are typically interpreted from averaged waveforms and therefore provide only an indirect view of trial‐level discriminability (i.e., how separable deviant and standard responses are on single trials). In contrast, EEGNet‐based decoding provides an estimate of single‐trial discriminability by quantifying how accurately deviant‐ versus standard‐evoked activity can be classified across individuals and task contexts (Lawhern et al. 2018). The finding that decoding accuracy was more strongly associated with behavioural efficiency in the active condition is consistent with accounts in which explicit task engagement increases the behavioural relevance and reliability of deviance‐related neural signals, strengthening brain–behaviour coupling (Sussman et al. 2002; Feldman and Friston 2010; Lindsay 2020). This single‐trial perspective also helps explain the comparatively lower decoding accuracy observed during passive listening: although deviance‐related responses are reliably elicited in passive paradigms, they are often smaller and more variable, which may limit fine‐grained trial‐level distinctions (Pakarinen et al. 2007). In the absence of explicit task demands, auditory processing is expected to be dominated by more automatic regularity‐based mechanisms, which may yield less distinctive trial‐level patterns than during active task performance (Chennu et al. 2013). In this sense, EEGNet provides a complementary window onto single‐trial discriminability that is important for understanding individual differences in performance efficiency.

At the group level, the high‐efficiency group showed higher deviant–standard decoding performance during the active task. This pattern aligns with the precision hypothesis in predictive coding, which proposes that task engagement may increase the precision‐weighting of deviance‐related prediction‐error signals (Feldman and Friston 2010). From this perspective, higher‐efficiency performers may engage more effective task‐related allocation, resulting in more discriminable deviant representations at the single‐trial level. This active‐specific pattern is consistent with top–down attention models that modulate sensory gain and improve signal‐to‐noise. For example, the normalization model (Reynolds and Heeger 2009) and reports of reduced neural variability under attention (Arazi et al. 2017; Lindsay 2020) support this view. At the individual level, decoding analyses further showed that greater neural discriminability was continuously associated with higher behavioural efficiency, indicating tighter brain–behaviour coupling under goal‐directed task demands.

Taken together, these findings suggest that the brain–behaviour relevance of MMN‐ and P3b‐related measures for individual differences depends on task context (Choi et al. 2014; Rutiku et al. 2024). By integrating EEGNet‐based decoding with ES, the current study indicates that active‐task responses yield stronger single‐trial discriminability that more closely tracks individual performance than passive‐listening responses. This task‐dependent strengthening of neural–behavioural coupling highlights how explicit engagement increases the behavioural relevance of deviance‐related activity. It extends prior ERP work by emphasizing trial‐level representational fidelity as a key dimension for understanding individual variability.

Several limitations should be noted when interpreting the present findings. First, because MMN was measured in a passive task and P3b was measured in a separate active task, we could not assess MMN and P3b within the same experimental context. Therefore, comparisons between MMN‐ and P3b‐related effects are limited. In addition, although the sample size was sufficient to detect medium‐to‐large effects, the male‐only and relatively homogeneous sample limits generalizability and may reduce sensitivity to more subtle associations. Finally, our decoding analyses were restricted to stimulus‐locked EEG responses and therefore may miss later and sustained decision‐related processes (e.g., extended integration, response preparation, and post‐decision activity) that could also drive individual differences in performance. Future work using longer‐latency and time‐resolved analyses, alternative decoding models, computational modelling, and complementary modalities such as MEG may further clarify how automatic regularity‐based processing and task engagement jointly shape perceptual decisions and individual differences in performance.

## 5 Conclusion

As demonstrated in the present study, comparisons of single‐trial decoding between active and passive conditions indicate that task engagement is associated with higher neural discriminability and stronger brain–behaviour coupling. Single‐trial decoding provided complementary sensitivity to individual differences that was not readily apparent from averaged ERP components alone, supporting its utility for characterizing variability in auditory cognition. Together, these findings provide a framework for linking task‐dependent neural discriminability to behavioural outcomes and motivate future applications in populations where quantifying individual differences in auditory processing is especially important.

## Author Contributions

**Zhaonan Ma:** conceptualization, data curation, formal analysis, methodology, software, validation, visualization, writing – original draft, writing – review and editing. **Xiaoyu Wang:** conceptualization, formal analysis, methodology, supervision, writing – review and editing. **Xiao Yang:** data curation, investigation, writing – review and editing. **Chao Guo:** investigation, methodology, writing – review and editing. **Tommi Kärkkäinen:** investigation, resources, supervision, writing – review and editing. **Fengyu Cong:** conceptualization, funding acquisition, resources, supervision, writing – review and editing.

## Funding

This work was supported by China Scholarship Council, 202106060036.

## Conflicts of Interest

The authors declare no conflicts of interest.

## Data Availability

The data supporting the findings of this research are available on request to the corresponding author, pending a formal data‐sharing agreement and approval from the local ethics committee.
